# Tired and out of control? Effects of total and partial sleep deprivation on response inhibition under threat and no-threat conditions

**DOI:** 10.1093/sleep/zsae275

**Published:** 2024-11-23

**Authors:** Arne Nieuwenhuys, Corey G Wadsley, Robyn Sullivan, John Cirillo, Winston D Byblow

**Affiliations:** Movement Neuroscience Laboratory, Department of Exercise Sciences, University of Auckland, Auckland, New Zealand; Department of Human Physiology, University of Oregon, Eugene, USA; Movement Neuroscience Laboratory, Department of Exercise Sciences, University of Auckland, Auckland, New Zealand; Discipline of Physiology, University of Adelaide, Adelaide, Australia; Movement Neuroscience Laboratory, Department of Exercise Sciences, University of Auckland, Auckland, New Zealand

**Keywords:** sleep restriction, anxiety, stress, selective stopping, performance

## Abstract

**Study Objectives:**

Sleep deprivation may impair top-down inhibitory control over emotional responses (e.g. under threat). The current study examined the behavioral consequences of this phenomenon and manipulated the magnitude of individuals’ sleep deficit to determine effect thresholds.

**Methods:**

Twenty-four healthy human participants were provided with 0, 2, 4, and 8 hours of sleep opportunity and, subsequently, performed a bimanual anticipatory response inhibition task under threat and no-threat conditions. Behavioral responses (button presses) and surface electromyography (EMG) from task effectors were collected to examine going and stopping processes.

**Results:**

Bayesian analyses revealed that compared to 8 hours of sleep, go-trial accuracy was reduced with 0 hours of sleep. Stopping speed was reduced with 0 and 2 hours of sleep, as evidenced by longer stop-signal delays, but only in a selective stopping context. None of the outcome measures were impacted by 4 hours of sleep. Under threat, go-trial accuracy was maintained, while responses were slightly delayed and characterized by amplified EMG bursts. Stopping speed was increased under threat across both stop-all and selective stopping contexts. No evidence was observed for interactions between sleep and threat.

**Conclusions:**

Sleep deprivation negatively affected response inhibition in a selective stopping context, with stopping speed reduced following a single night of ≤2 hours of sleep. Performance-contingent threat improved response inhibition, possibly due to a prioritizing of stopping. No evidence was observed for increased threat-related responses after sleep deprivation, suggesting that sleep deprivation and threat may impact inhibitory control via independent mechanisms.

## Graphical Abstract

**Graphical Abstract:**
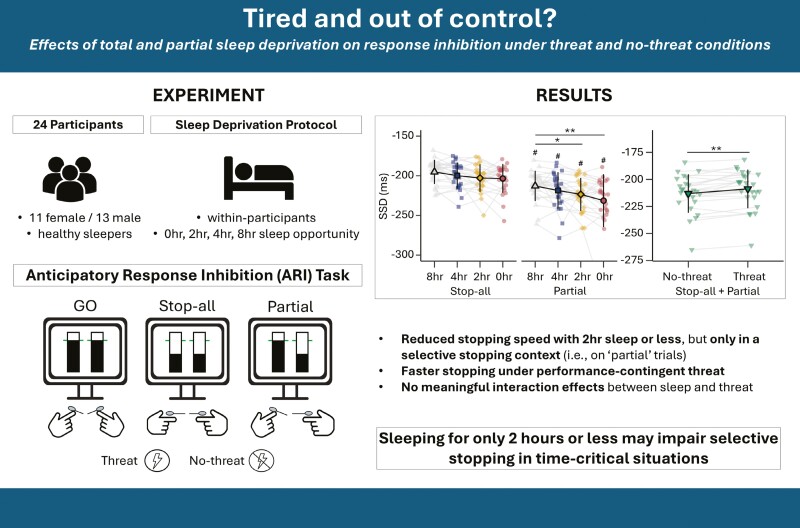

**Statement of Significance**

Response inhibition is required for adaptive responding in many daily life situations. Insufficient sleep may pose a risk to inhibitory functioning especially in high-threat situations, due to amplified hyper-limbic responding. The current study investigated the effects of sleep deprivation and threat on response inhibition across different amounts of sleep deprivation and found that a single night of sleeping 2 hours or less impairs response inhibition in selective but not nonselective stopping contexts—where specific components of a multi-component action need to be stopped, while other components may continue. No evidence was observed for increased threat-related responses after sleep deprivation. Findings bear implications regarding the extent of sleep deprivation and specific contextual factors that pose a meaningful risk to individuals’ inhibitory performance.

Sleep is often recognized to be the most important recovery mechanism of the human body [1]. Yet, the prevalence of insufficient sleep is high, with a third of the population sleeping less than 7 hours per night [2], and many people experiencing restricted sleep in the context of work, travel, and other aspects of daily life [3]. Insufficient sleep impairs brain functioning and results in well-documented cognitive deficits in attention and alertness [4, 5]. Pertinent to the present study, an important cognitive function that may be affected by sleep, is the ability to withhold or stop a preplanned or ongoing action—typically referred to as “response inhibition” [6]. Response inhibition supports social functioning in daily life and is crucial for adaptive responding in high-stress situations, for example, in managing social conflict [7], or law enforcement [8]. In these and other situations, failure to inhibit undesired or impulsive actions can have serious consequences, including the potential to pose a risk to health or life. There is mounting evidence that sleep deprivation may impair top-down inhibitory control over emotional responses [9]. The present study set out to investigate the effects of sleep deprivation on response inhibition in threat and no-threat conditions and manipulated the magnitude of individuals’ sleep deficits to determine effect thresholds.

Response inhibition may be implemented in nonselective and selective stopping contexts [10]. In a *nonselective stopping* context, response inhibition is implemented to stop all components of an action. For example, when a pedestrian attempts to cross a road but suddenly notices an approaching car, response inhibition is implemented to prevent stepping onto the road completely and avoid a possible collision. In more complex *selective stopping* contexts [11], response inhibition is implemented to only stop an action in relation to a specific subset of stimuli (i.e. “stimulus-selective” stopping) or to stop a subset of a multi-component action (i.e. “response-selective” stopping). For example, when the driver of a car attempts to change lanes but suddenly notices another car emerging from their blind spot, response inhibition is used to prevent the intended steering motion, but other components involved with driving the vehicle may continue.

Successful application of response inhibition across selective and nonselective stopping contexts involves distinct neural processes. Response inhibition is supported by a putative neural network comprised of cortico-basal-ganglia “hyperdirect” and “indirect” pathways [12]. Fast global suppression of motor output is achieved via the hyperdirect pathway [13], while targeted yet slower suppression of motor output is achieved via the indirect pathway [14]. Response-selective stopping (henceforth referred to as “selective stopping”) can be achieved via both pathways but has been shown to often involve at least some degree of global inhibition, such that motor output is initially suppressed across all effectors before being reinstated in non-stopping effectors [10]. Consequently, selective stopping often results in an observable response delay in nonstopped effectors—a phenomenon that is typically referred to as the “stopping-interference effect” [15].

Insufficient sleep is believed to impair cognitive control due to decreased stability in arousal levels, which causes reciprocal inhibition between on-task and off-task brain networks to become more variable [9]. This proposition is supported by research consistently associating sleep deprivation with reduced and often variable cognitive performance [5]. Sleep restriction (i.e. sleep durations shortened below the age-recommended range) has also been associated with decreased cognitive functioning in specific domains, including inhibitory control [4]. The effect of sleep deprivation on response inhibition, however, is less clear. To date, only a handful of studies have considered the effects of insufficient sleep on response inhibition using recommended stop-signal task (SST [16]) or anticipatory response inhibition (ARI [17]) paradigms. Both SST and ARI paradigms make use of an explicit stop-signal that is presented following an initial go-signal and, as such, better capture response inhibition as a form of top-down inhibitory control than often-used go/no-go paradigms [18, 19]. Using the SST, Van Peer et al. [20] found no robust evidence for an effect of sleep restriction (i.e. three nights of 5 vs. 8 hours of sleep) on individuals’ stop-signal reaction time, which is a measure that is generally taken to reflect the speed of the underlying inhibitory process (i.e. “efficiency” of inhibition [21]). Zhao et al. [22] found longer stop-signal reaction times after 24 hours (one night) of total sleep deprivation, whereas Kusztor et al. [23] found no robust evidence for an effect of total sleep deprivation on stop-signal reaction times—although sleep deprivation did impact Go trial success rates and more strategic (top-down) aspects of inhibitory control [23]. As such, more research using recommended SST and ARI task paradigms is needed to determine if and when insufficient sleep impairs response inhibition in the form of top-down inhibitory control.

Neuroimaging studies suggest that sleep-deprived individuals may be at risk of reduced top-down inhibitory control, especially in emotional circumstances [9]. Under high stress, activation of the salience network increases attention to threat [24], fast-tracks the processing of threat-related information [25], and increases cortico-spinal excitability [26]. These responses are believed to be functional in that they facilitate quick stimulus-driven responses to threats (e.g. fight or flight) but often interfere with performance on tasks that require efficient goal-directed control [27, 28]. In a seminal study, Yoo et al. [29] showed that, compared with normal sleep, individuals who underwent one night of total sleep deprivation showed an amplified hyper-limbic response when exposed to emotional content (aversive pictures). This effect has been replicated in later studies and extended to also include amplified reactivity to pleasure-evoking stimuli [9, 30]. Examinations of downstream behavioral effects, however, are scarce and, thus far, show mixed results. That is, total sleep deprivation and habitual (i.e. chronic) sleep restriction have been associated with greater impulsivity and risk-taking behavior, specifically in negative affective circumstances [31, 32], but three nights of 5 versus 8 hours of sleep did not exacerbate effects of threat on response inhibition [20]. In short, the extent to which sleep deprivation impairs top-down inhibitory control over emotional responses may be dose-dependent, and sleep loss either needs to be more severe (i.e. total sleep deprivation) or accumulated over a longer period of time, for behavioral effects to be observed (cf. [20],).

The present study aimed to elucidate the effects of sleep deprivation on response inhibition under threat and no-threat conditions and to determine effect thresholds by systematically manipulating the magnitude of participants’ sleep deficit (i.e. one night of 0, 2, 4, and 8 hours of sleep opportunity). Effects of sleep and threat on response inhibition were investigated using a bimanual ARI task with stop-all and partial trials, to examine effects across selective and nonselective stopping contexts. The ARI task was selected because it provides a robust assessment of response inhibition while minimizing options for strategic responding (e.g. “strategic slowing” [19]). A performance-contingent threat of shock protocol was implemented to assess the effects of threat on response inhibition [33]. Electromyographical (EMG) recordings were collected from task effectors to gain insight into neurophysiological processes underlying behavioral responses. Four primary hypotheses were tested: (1) Detrimental effects of insufficient sleep on behavioral going and stopping (e.g. reduced go success rates, decreased stopping speed) would increase with increasing sleep deficit, owing to cumulative costs of wakefulness on cognitive functioning [9, 34]. (2) Effects of sleep on response inhibition would be exacerbated in a selective stopping context, due to the greater attentional and cognitive demand associated with selective stopping [10, 11]. (3) Performance-contingent threat would lead to more potentiated go responses as observed from EMG recording, impaired response inhibition, and greater stopping-interference, due to increased action readiness and reduced cognitive control in the context of acute threat [20, 24, 28]. (4) Sleep deprivation but not sleep restriction would exacerbate observed behavioral effects of threat (e.g. amplified initiation of motor responses, more severely impaired response inhibition), reflecting reduced top-down inhibitory control over emotional responding [9, 29, 31].

## Methods

### Participants

Based on an a priori power analysis computed in G*Power [35], the current study required 24 participants to detect an estimated medium-sized interaction effect (*f* = 0.25 [29, 31]) with 80% power and alpha 0.05, using analysis of variance (ANOVA) with a 4 × 2 repeated measures design. A total of 43 participants, recruited via study advertisements posted on public and online notice boards around the university, were screened for participation. As part of the screening process, participants completed the Pittsburgh Sleep Quality Index (PSQI [36]), Holland Sleep Disorder Questionnaire (HSDQ [37]), State-Trait Anxiety Inventory A-Trait Scale (STAI A-Trait [38]), Barratt Impulsiveness Scale (BIS [39]), short version of the Edinburgh Handedness Inventory (EHI [40]), and a custom safety screening and substance dependence checklist. Exclusion criteria were existing sleep problems, as indicated by a score >6 on the PSQI ([36]; cf. [41]) or a score >2.02 on the HSDQ [37], any self-disclosed health condition that precluded safe use of experimental procedures (e.g. epilepsy, pregnancy), and any self-reported, currently existing substance dependency, operationalized as habitual intake of >2 caffeinated/alcoholic beverages per day, any smoking, any psychoactive medication/drugs, or use of sleep supplements/medication. Twelve participants were excluded based on these criteria (existing sleep problems, *n* = 6; existing sleep problems combined with other health conditions and/or substance dependence, *n* = 6), two participants withdrew participation prior to scheduling experimental sessions (lack of availability), and a further five participants did not complete all experimental sessions (change in personal circumstances, lack of availability). Relevant trait characteristics of the remaining 24 participants (mean age = 26.64 ± 5.51 years; 11 female, 13 male; 22 right-handed, 2 left-handed) are listed in Table 1. Participants were informed about the study protocol and provided written informed consent prior to starting the first experimental session. Participants were offered NZ$400 (NZ$100 per session) in return for their participation. The study protocol received ethical approval from the University of Auckland Human Participants Ethics Committee (reference number: UAHPEC20597) and was conducted in accordance with the Declaration of Helsinki [42].

**Table 1.** Participant Descriptives (*n* = 24)

	Median	Range
PSQI (0–21)	4	1–6
HSDQ (1–5)	1.5	1.16–1.94
STAI A-trait (20–80)	35.5	23–53
BIS-11 (30–120)	62	49–75

PSQI, Pittsburgh Sleep Quality Index; HSDQ, Holland Sleep Disorder Questionnaire; STAI A-Trait, State-Trait Anxiety Inventory A-Trait scale; BIS-11, Barratt Impulsiveness Scale 11.

### Study design and procedure

The study had a 4 × 2 (sleep × threat) repeated measures design, featuring a within-participant manipulation of sleep (i.e. 0, 2, 4, and 8 hours of sleep opportunity; see “Sleep Deprivation Protocol” section, below) and a within-participant manipulation of threat (no-threat vs. threat; see “experimental task” section, below). Participants attended four overnight experimental sessions (one for each sleep condition) scheduled at least one week apart to allow for washout. In the week leading up to each session, participants self-monitored their sleep on a day-to-day basis, using the Consensus Sleep Diary [43]. On the day of the session, in case sleep had not returned to baseline and/or external factors had caused sleep to deviate >2 hours from participants’ self-reported habitual bed/rise times (as reported on the PSQI), the session was canceled and rescheduled to a later date.

#### Sleep deprivation protocol

The sleep deprivation protocol took place at the university, in a large laboratory environment consisting of an open workspace/living area, kitchenette, and adjacent bedroom and laboratory spaces used for behavioral testing. Two trained research assistants (overnight supervisors) were present at all times. Participants reported to the laboratory at 10:00 pm and were provided with 0, 2, 4, or 8 hours of sleep opportunity from 11:00 pm (order randomized across participants). During their allotted sleep time, participants slept in a private bedroom immediately adjacent to the central laboratory space. Personal devices (phone, laptop, etc.) were not allowed inside the bedroom and while a small bedside lamp was provided for participants’ comfort, windows were blinded to prevent any external light from entering. Accumulated sleep time was monitored objectively using a wrist-worn research-grade accelerometer (GENEActiv, Activinsights Ltd., Kimbolton, UK) and subjectively using the Consensus Sleep Diary ( [43]; completed upon awakening). Accelerometer data were collected in 60-second bins and analyzed offline using GENEACTiv software version 3.2 and the GENEActiv R Markdown Sleep Report analysis tool version 1.0.4 (Activinsights, Ltd., 2023). Total sleep time (TST) was the only variable of interest. During their allotted wake time, participants remained in the central laboratory space together with both research assistants, engaging in conversation, reading, playing board games, or engaging in light physical activity (walking), as required, to help them stay awake. Participants could drink water/herbal tea and eat crackers (no sugar) when hungry. Otherwise, no food or sugar/caffeinated beverages were provided. The ambient temperature and lighting across the laboratory were kept constant at 19–22°C and ~150–200lux, respectively. Participants consumed a standardized light breakfast at 07:30 am. To verify the effects of the different sleep conditions on subjective and objective sleepiness, participants completed the Karolinska Sleepiness Scale and Global Vigor and Affect Scale (KSS [44]; GVA [45]) and performed a 10-minute Psychomotor Vigilance Task (PVT [46]), at 10:00 pm (baseline) and each subsequent hour of wake time, with the last assessment taking place at 08:00 am, immediately prior to starting the experimental session. PVT data was analyzed offline using a custom analysis script written in Python software. Error rate (lapses and false starts) and response speed (mean 1/RT) at 10:00 pm and 08:00 am, were selected as main outcome measures of interest based on [47].

#### Experimental task

**Figure 1.**
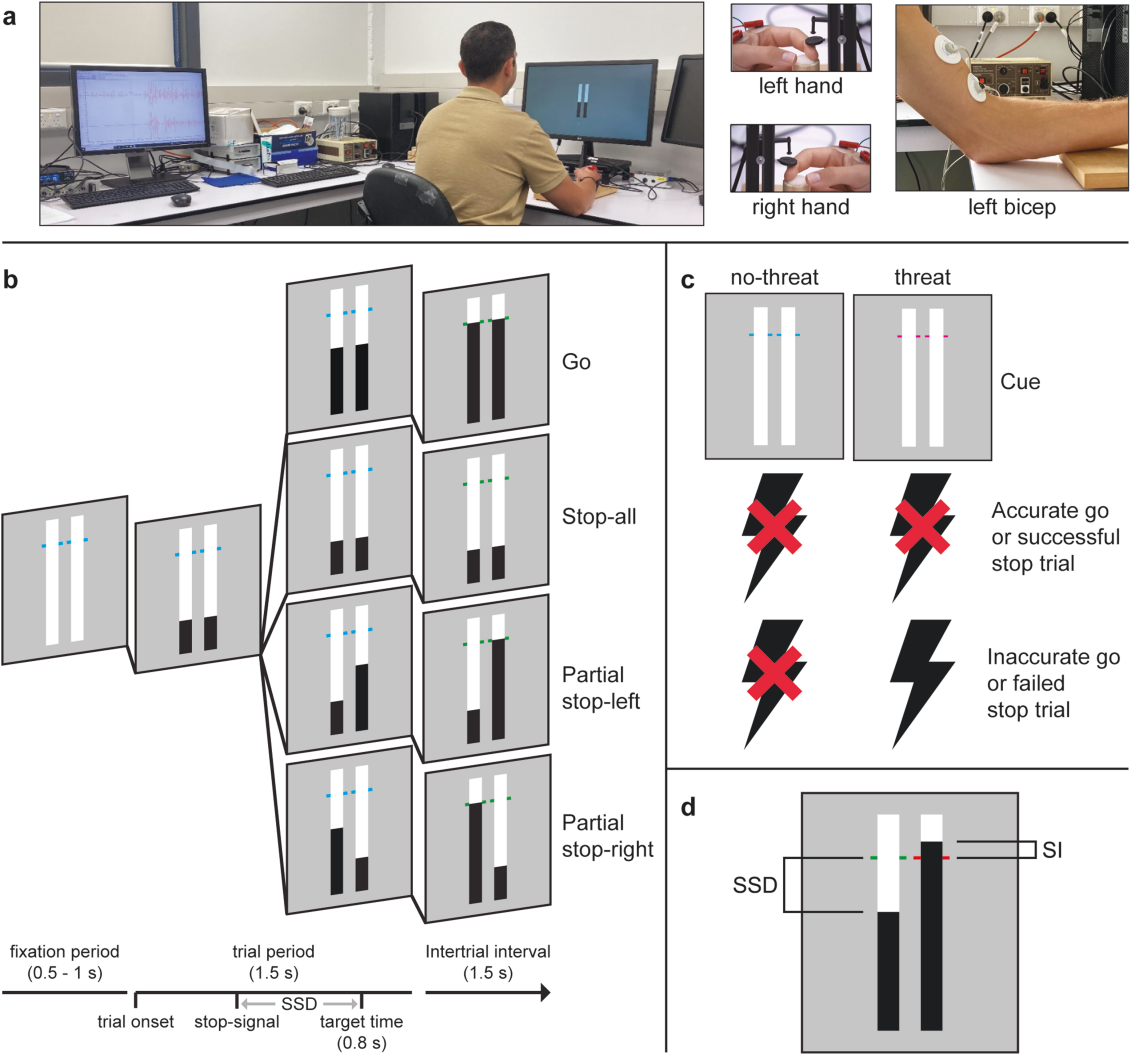
**(A)** Overview of the experimental set-up. **(B)** Trial flow for each trial type. **(C)** Threat and no-threat conditions (indicated by the color of the target line) and associated shock contingencies. **(D)** Partial stop-left trial indicating stop signal delay (SSD; left bar) and stopping interference (SI; right bar).

The experimental task for each session started at 08:15 am, immediately following the sleep deprivation protocol. A bimanual version of the Anticipatory Response Inhibition (ARI) task was used to assess participants’ response inhibition in selective and nonselective stopping contexts [10]. Participants were seated at a desk, facing an LG-24GL600F-B computer display (refresh rate 144 Hz; viewing distance ~60 cm). Participants had their lower arms resting on the desk and their index fingers resting on small blocks that were positioned shoulder-width apart in front of them. Block height was adjusted to minimize postural activity as observed from EMG recording of the task-relevant (left and right) first dorsal interosseous (FDI) muscle. Mechanical response switches were positioned immediately above both index fingers, allowing participants to interact with the task through sagittal index finger abduction. The ARI task was programmed with the Selective Stopping Toolbox [19] using PsychoPy software (v2022.1.4) [48]. All task equipment was synchronized using a custom Arduino Leonardo response board. Figure 1 shows an overview of the experimental task and setup.

The default task display consisted of a gray background with two white bars (10 cm high, 1.5 cm wide). A colored target line (cyan or magenta, depending on the threat condition) was positioned at 80% of the bars’ original height. Each trial consisted of a preparation period (500–1000 milliseconds), a trial period (1000 milliseconds), and a feedback period (1200 milliseconds). The trial start was indicated by both bars appearing to “fill” from the bottom upwards. Bars took exactly 1000 milliseconds to fill completely. The majority of trials were “go” trials during which the objective was to lift the index fingers to press the corresponding mechanical switches to stop the left and right bar from filling as close as possible to the target lines (i.e. target response time = 800 milliseconds). In some trials, however, either both bars (“stop-all” trial; nonselective stopping) or only the left or right bar (“partial” trial; selective stopping) would automatically stop filling before reaching the target line. In these cases, the objective was to respond only with the index finger(s) corresponding to the bar which continued rising. The initial delay for this ‘stop-signal’ to occur (stop signal delay [SSD]), was set at 250 milliseconds prior to the target line and this was subsequently adjusted upward or downward on a trial-by-trial basis in steps of 35 milliseconds (corresponding to ~5 frames), based on stopping success. For each trial type separately, the SSD was adjusted downward (less time before the target line) in case of successful stopping and upward (more time before the target) in case of unsuccessful stopping, thus ensuring an average stopping success of ~50% across both stop-all and partial trials. To encourage accurate responding throughout the task, points were awarded based on relative response times (RT_rel_; relative to the target line) and stopping success. Points were indicated by changing the color of the target line during the trial feedback period (i.e. immediately upon trial completion); with “green” = 100 points (RT_rel_ < 25 milliseconds or successful stop), “yellow” = 50 points (RT_rel_ = 26–50 milliseconds), “orange” = 25 points (RT_rel_ = 51–75 milliseconds), and “red” = 0 points (RT_rel_ > 75 milliseconds or failed stop).

During the experimental task, EMG recordings were collected from the task-relevant (left and right) FDI muscle. Ag-AgCl surface electrodes (CONMED) were placed in a standard belly-tendon montage and a ground electrode was positioned on the posterior surface of the left hand. EMG activity was amplified (×1000), bandpass filtered (10–1000 Hz), and sampled at 2500 Hz using a CED data acquisition unit (MICRO1401mkII; Cambridge Electronic Design) and recorded using Signal software (v7.05).

A performance-contingent *threat-of-shock* protocol was implemented to investigate the effects of threat on response inhibition [33]. In the Threat condition, participants received a mild electric shock (uncomfortable ‘pinch’) immediately upon completion of each trial that resulted in RT_rel_ > 50 milliseconds or failed stop, thus ensuring that an aversive consequence of inaccurate responding was implemented across all trial types [20]. Shocks (single-pulse, 200 μs, 400 V, 15–90 mA) were delivered using a Digitimer DS7A electrical stimulator, which was interfaced with the task computer and connected to the distal end and center of participants” left biceps brachii muscle via two Ag-AgCl Suretrace RTL 1800C-050 electrodes. Prior to starting the task, shock intensity (mA) was adjusted such that for each individual participant, subjectively perceived intensity equated to a score of “4” (“*uncomfortable*”) on a 10-point visual analog rating scale (“*no distress*”-“*unbearable distress*”). During the task, the perceived intensity of the shocks was monitored and objective shock intensity was adjusted as required, to ensure that perceived intensity remained at and did not exceed a score of 4 (i.e. did not become “*painful*”). The possibility of receiving an electrical shock ensured that, in the Threat condition, performance occurred under increased levels of anxiety [33]. In the No-Threat condition, no shocks were delivered.

Two practice blocks of 36 go trials—one in the threat condition and one in the no-threat condition were performed prior to the ARI task. This was done to familiarize with the task and establish a predominant go-response. The ARI task required a further 14 blocks of 36 experimental trials each (i.e. 504 trials in total: 336 go trials, 56 stop-all trials, and 112 partial trials). The trial type was randomized within blocks, but such that each block always started with a go trial. The threat was manipulated on a block-by-block basis, with Threat and No-Threat blocks in alternating order and block type being indicated via the color of the target line (i.e. cyan or magenta). The order of threat conditions (i.e. threat first or no-threat first) and threat-color coupling was counterbalanced across participants. A 1-minute break was taken after each block. On average, each experimental session lasted ~1.5 hours.

### Dependent variables

#### Manipulation check

At the end of the experimental session, participants completed an exit questionnaire to determine subjective responses to the threat manipulation [20]. Using a 9-point Likert scale, the questionnaire assessed participants’ self-perceived *task motivation* (“not motivated at all” – ‘very motivated’) and, using pictorial Self-Assessment Manikins [49], perceived *valence* (“pleasant”-‘unpleasant’), *arousal* (“excited”-‘calm’) and *dominance* (“controlled”-“in control”), in the threat and no-threat condition.

#### Behavioral data

Behavioral data were directly taken from the ARI task and analyzed offline using custom scripts in Python. *Trial success* was determined as the percentage of trials with correct responses within the trial period for go trials (both hands), correct withholding of responses for stop-all trials (both hands), and correct withholding and responding for partial trials (signaled and non-signaled hand, respectively). *Response times* on go trials (Go RT, in ms) were calculated by taking the mean recorded RT across both hands and subtracting the target RT of 800 milliseconds. Values below and above zero indicate early and late responses, respectively. Mean *SSD* (in milliseconds) was taken as the primary measure of stopping speed [19], with lower SSDs indicating less time required for stopping. *Stopping Interference* (SI) was calculated by subtracting the mean RT on go trials from the mean RT of the responding hand on successful partial trials [50]. Greater SI values reflect greater SI.

#### Electromyographical data

EMG data were preprocessed using custom scripts in MATLAB and subsequently analyzed in Python. Based on [51], all trial data was bandpass filtered (20–250 Hz) using a second-order Butterworth filter, resampled to 500 Hz, and smoothed by taking the root mean square over a 50 milliseconds sliding window. Data were then normalized to baseline (200–400 milliseconds after trial onset) and *z*-scored for each hand and participant separately. Response-related EMG bursts were identified within a time window of 400–1000 milliseconds from trial onset [50]. Briefly, burst onset and offset were determined by starting within a peak of EMG activity and taking the time of the first data point within a 10 milliseconds window in either direction where the level fell below a threshold of 1.2 z. The peak gradient of the EMG burst was defined as the maximum value of the differentiated signal between burst onset and peak. Data were converted back to raw mV prior to statistical analyses, with dependent EMG variables including *baseline EMG* (mV), *burst-onset* (ms), *burst-amplitude* (mV), and *burst*-*gradient* (mV/ms). Due to a low number of bursts detected in relation to successful stopping, burst analyses focused on successful responding on-go trials and partial trials only.

### Statistical analysis

Data were analyzed with Bayesian analyses of variance (ANOVA) and *t*-tests, using JASP software (Version 0.17.2; JASP Team, 2023) with default prior distributions [52]. Prior to analyses, the normality of the data and model-averaged residual plots were visually inspected. Analyses of variance included “participant” as a random intercept and applied random slopes (fitted across 10 000 iterations) for each within-participant factor. Interaction effects were determined by comparing models with the interaction term to models without the interaction term. Evidence for main and interaction effects was interpreted using the Bayes factor (BF_10_ ± percentage error), with values <1 reflecting evidence for the null hypothesis and values >1 reflecting evidence for the alternative hypothesis. As per [52], effects with BF_10_ < 0.3 and BF_10_ < 0.01 indicate moderate and strong evidence for the null hypothesis; 0.3 ≤BF_10_ ≤ 3 indicate inconclusive evidence, and BF_10_ > 3 and BF_10_ > 10 indicate moderate and strong evidence for the alternative hypothesis. Significant main and interaction effects were followed up using Bayesian *t*-tests for each pairwise comparison, with posterior odds (*O*_post_) corrected for multiple comparisons using the Westfall approach [53].

To verify the effectiveness of the sleep deprivation protocol, TST was examined using a one-way RM ANOVA with four levels of sleep. KSS scores, PVT error rate and response speed, at baseline (10:00 pm) and immediately prior to starting the experimental task (08:00 am), were examined with 4 × 2 (sleep × time) RM ANOVAs. As a manipulation check of threat, all variables included in the exit questionnaire (i.e. motivation, valence, arousal, and dominance scores) were examined using 4 × 2 (sleep × threat) RM ANOVAs. For the behavioral data, go trial success, go response times, and SI, were examined using 4 × 2 (sleep × threat) RM ANOVAs, while stopping success and stop-signal delay on stop-all and partial trials, were examined using 4 × 2 × 2 (sleep × threat × trial type) RM ANOVAs. For the electromyographical data, baseline EMG was examined using a 4 × 2 (sleep × threat) RM ANOVA, while burst-onset, burst-amplitude, and burst-gradient, on successful go and partial trials, were examined with 4 × 2 × 2 (sleep × threat × trial type) RM ANOVAs. The hypothesized increase in threat-related responses under sleep deprivation is reflected in the interactions between sleep and threat.

## Results

### Manipulation checks

A full description of the results for all manipulation checks is included in Supplementary Material. Data from the sleep deprivation protocol are presented in Supplementary Tables S1 and S2, Figure S1. Data from the exit questionnaire (manipulation check of threat) are presented in Supplementary Table S3. For TST, there was a strong main effect of sleep (BF_10_ = 2.97 × 10^54^ ± 0.49%), indicating that all sleep conditions were effectively separated (O_post_’s ≥ 9.63 × 10^6^). Subjective sleepiness was higher and vigor lower with less sleep, with moderate to strong differences between all conditions (O_post_’s ≥ 6.40), apart from the 0 and 2 hours conditions, which did not meaningfully differ from each other (O_post_’s ≤ 0.75; Supplementary Table S1). PVT error rate was higher and PVT response speed was lower with 0-hour sleep than with 2, 4, and 8 hours sleep (O_post_’s ≥ 14.31; O_post_’s ≥ 27.96), and with 2 hours sleep than with 8 hours sleep (O_post_ = 3.72; O_post_ = 80.70; Supplementary Figures S1A and S1B). Response speed was also lower with 4 hours of sleep than with 8 hours of sleep (O_post_ = 15.67). Subjective sleep estimates as recorded during the week preceding each experimental session showed no meaningful main effects of sleep and in most cases reflected evidence for the null hypothesis (see Supplementary Table S2 for an overview), indicating that effects of sleep as observed in the present study are unlikely to have been influenced by variability in sleep that occurred outside of the experimental sessions. Regarding the manipulation check of threat, data from the exit questionnaire indicate that during the ARI task, motivation was higher, valence more negative, and arousal higher, in the Threat than in the no-threat condition (BF_10_’s > 1744.28; Supplementary Table S3). The main effect of sleep remained inconclusive (0.32 ≤ BF_10_ ≤ 0.70) and the interaction between sleep and threat reflected evidence for the null hypothesis (0.15 ≤ BF_10_ ≤ 0.26).

### Behavioral data

**Figure 2.**
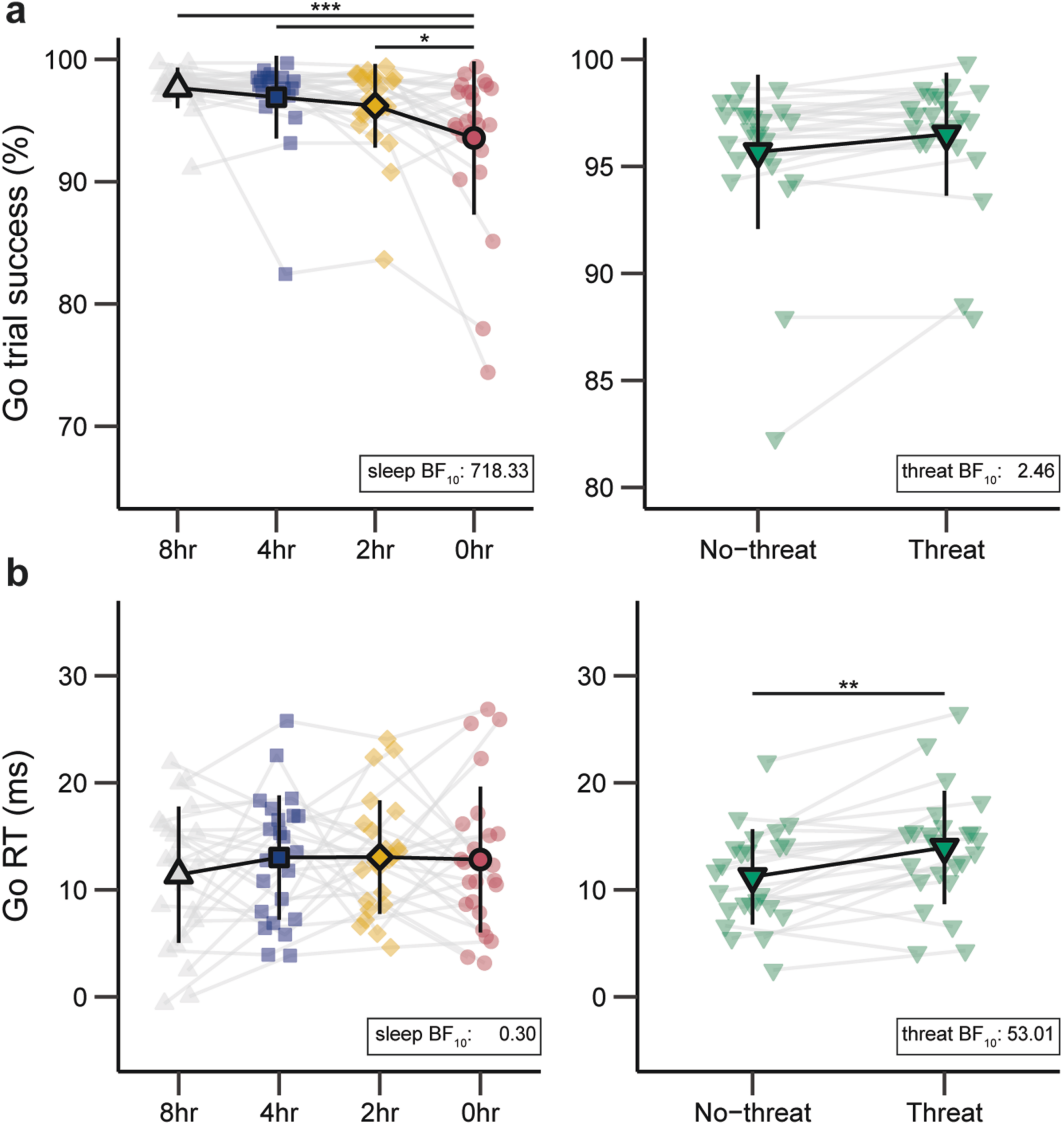
Go trial success rate (**A**) Go trial response time (Go RT; (**B**) across sleep (8, 4, 2, and 0 hours) and threat (No-threat, Threat) conditions. Error bars indicate 95% confidence intervals. * O_post_ / BF_10_ > 3; ** O_post_ / BF_10_ > 10; *** O_post_ / BF_10_ > 100.

**Figure 3.**
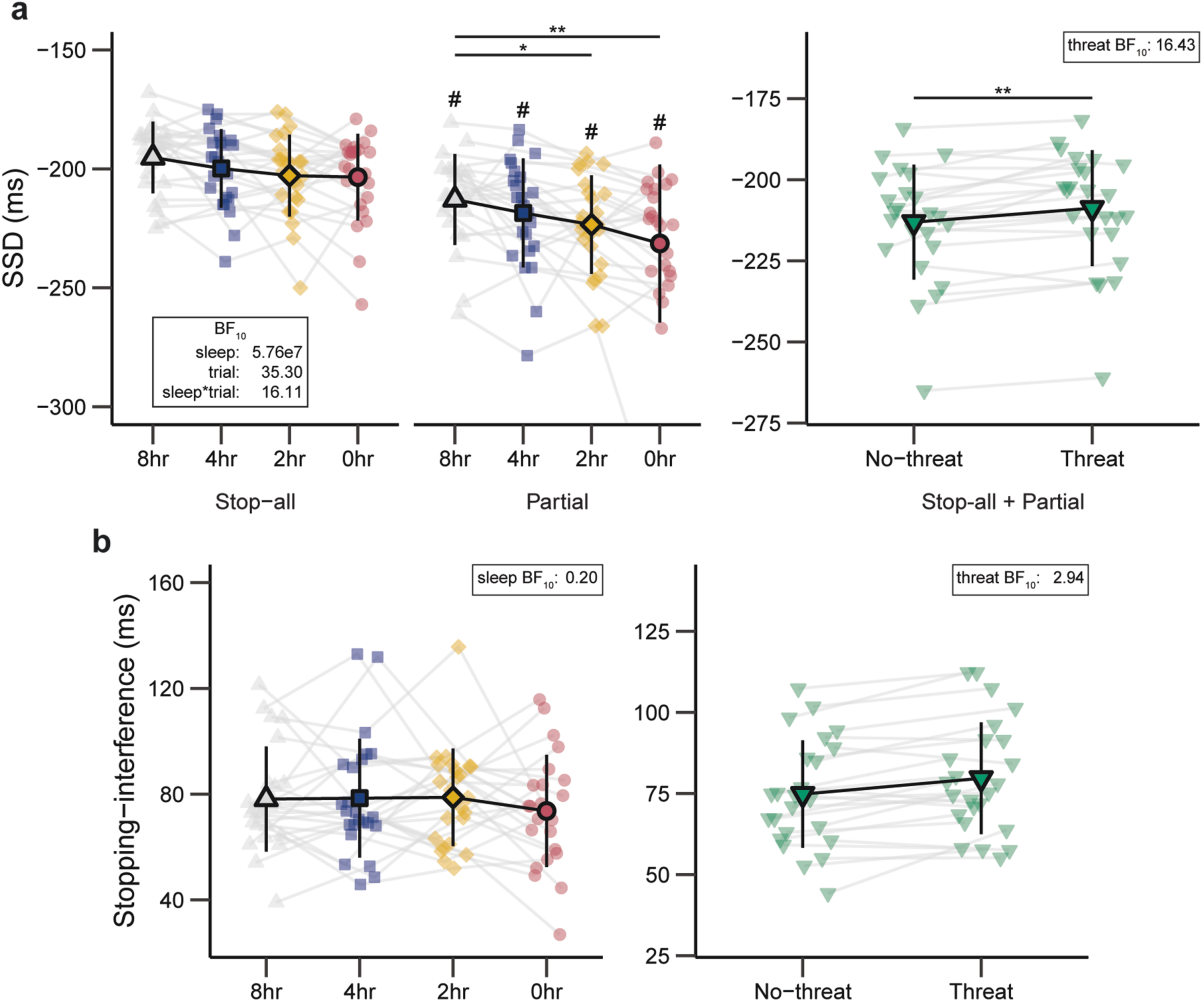
Stop-signal delay (SSD) (**A**) and Stopping-interference (**B**) across sleep (8, 4, 2, and 0 hours) and threat (no-threat, threat) conditions. Error bars indicate 95% confidence intervals. * O_post_ / BF_10_ > 3; ** O_post_ / BF_10_ > 10; ^#^ = SSD meaningfully longer for partial trials than for stop-all trials (i.e. main effect of trial type: O_post_ ≥ 3).

Behavioral data from the ARI task are presented in Supplementary Table S4 and Figures 2 and 3.

#### Go trial success rate and response times

For success rate, there was a strong main effect of sleep (BF_10_ = 718.33 ± 1.13%). The success rate with 0-hour sleep was lower than with 8 hours of sleep (O_post_ = 1291.44), 4 hours of sleep (O_post_ = 1048.57), and 2 hours of sleep (O_post_ = 9.17; Figure 2a). Success rates with 2 and 4 hours of sleep did not differ from 8 hours (O_post_’s ≤ 1.3), nor were they different from each other (O_post_ = 0.32). Evidence for the effect of threat (BF_10_ = 2.46% ± 2.52%) and the interaction between sleep and threat (BF_10_ = 0.53% ± 2.91%) was inconclusive. For response time, there was a strong main effect of threat (BF_10_ = 53.01% ± 2.74%). Response times were later in the Threat than in the no-threat condition (Figure 2B). The main effect of sleep (BF_10_ = 0.296% ± 11.38%) and the interaction between sleep and threat (BF_10_ = 0.06% ± 23.40%) both reflected evidence for the null hypothesis.

#### Stopping success rate, stop-signal delay, and stopping interference

 For all conditions, the mean success rate was close to 50% (range: 50.97%–53.87%; Supplementary Table S4), indicating that the staircase procedure worked effectively. There was a strong main effect of trial type (BF_10_ = 76228.68% ± 1.71%). The success rate was lower on partial trials (selective stopping) than on stop-all trials (nonselective stopping). The interaction between sleep and threat (BF_10_ = 0.09% ± 1.78%) and the three-way interaction between sleep, threat, and trial type (BF_10_ = 0.07% ± 3.77%) both showed evidence for the null hypothesis. Other main and interaction effects remained inconclusive (0.37 ≤ BF_10_ ≤ 0.98). For SSD, there were strong main effects of trial type (BF_10_ = 5.76 × 10^7^ ± 5.68%) and sleep (BF_10_ = 35.30% ± 5.68%), which were superseded by a strong trial type × sleep interaction (BF_10_ = 16.11% ± 8.18%). SSDs were longer (i.e. more time required for successful stopping) on partial trials than on stop-all trials (O_post_ = 2.804 × 10^43^; Figure 3A). Sleep deprivation impeded stopping speed in a selective (BF_10_ = 140.07% ± 0.78%) but not a nonselective (BF_10_ = 1.55% ± 0.68%) stopping context. On partial trials, SSDs with 0 and 2 hours of sleep were longer than with 8hr of sleep (O_post_ = 28.42 and O_post_ = 8.52; Figure 3A). Evidence for other comparisons remained inconclusive (O_post_’s ≤ 1.84). SSD analyses also revealed a strong main effect of threat (BF_10_ = 16.43% ± 6.29%). SSDs were shorter (i.e. less time required for successful stopping) in the Threat than in the no-threat condition (Figure 3A). The interaction between sleep and threat (BF_10_ = 0.13% ± 6.65%) and the three-way interaction between sleep, threat, and trial type (BF_10_ = 0.07% ± 15.52%) both showed evidence for the null hypothesis. The interaction between threat and trial type remained inconclusive (BF_10_ = 0.74% ± 11.25%). For SI, evidence for the main effect of threat was inconclusive (BF_10_ = 2.94% ± 2.00%), while the main effect of sleep (BF_10_ = 0.2% ± 1.08%) and the interaction between sleep and threat (BF_10_ = 0.07% ± 2.42%) both showed evidence for the null hypothesis (Figure 3B).

### Electromyographical data

**Figure 4.**
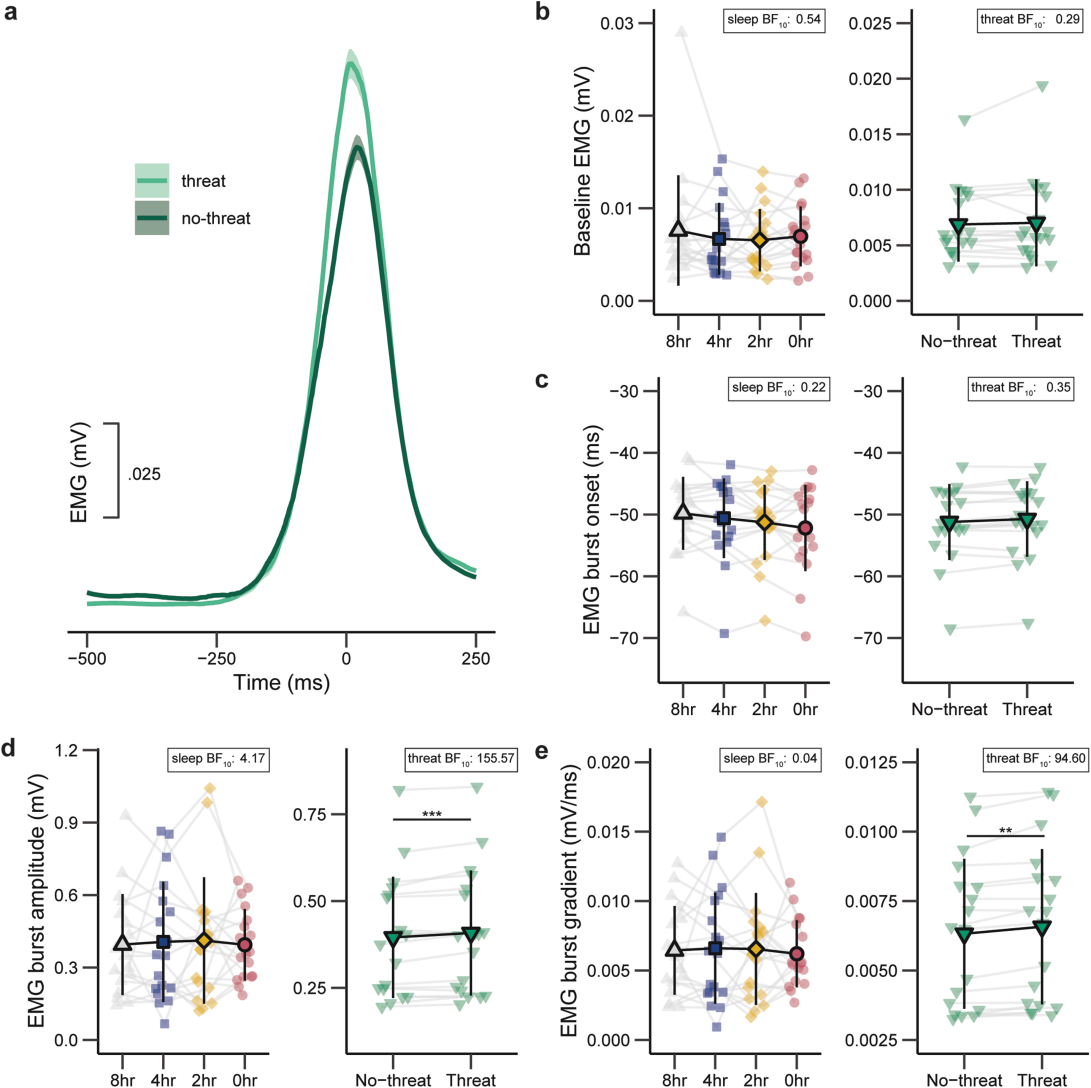
**(A)** Grand average electromyographical (EMG) trace on successful go- and partial-trials for the No-threat and Threat condition. **B-E:** Baseline EMG, EMG burst-onset, EMG burst-amplitude, and EMG burst-gradient, across sleep (8, 4, 2, and 0 hours) and threat (no-threat, threat) conditions. Error bars indicate 95% confidence intervals. ** BF_10_ > 10; *** BF_10_ > 100.

Electromyographical data are presented in Supplementary Table S5 and Figure 4. Complete EMG data from all four experimental sessions was available for 18 out of 24 participants due to technical issues. Post hoc analyses indicated that the behavioral results for this subsample are similar to that of the total sample, with all reported main effects of sleep and threat maintained (Supplementary Table S6).

#### Baseline EMG

For baseline EMG amplitude, evidence for the main effect of sleep (BF_10_ = 0.44% ± 17.67%) and interaction between sleep and threat (BF_10_ = 1.16% ± 24.76%) remained inconclusive, while the main effect of threat (BF_10_ = 0.27% ± 1.43%) reflected evidence for the null-hypothesis. In all conditions, baseline EMG was ≤0.008 mV, indicating that EMG burst analyses are unlikely to be confounded by differences in background EMG levels.

#### EMG burst analyses

For burst-onset, evidence for all main and interaction effects remained inconclusive (0.37 ≤ BF_10_ ≤ 0.62) or, in the case of the main effects of trial type and sleep, the interaction between trial type and threat, and the interaction between sleep and threat, showed evidence for the null hypothesis (BF_10_’s ≤ 0.27). For burst-amplitude, there was a strong main effect of threat (BF_10_ = 155.57% ± 24.39%), which was superseded by a strong interaction between trial type and threat (BF_10_ = 26.80% ± 24.69%). Across both go and partial-respond trials, burst amplitude was higher in the Threat than in the No-Threat condition. There was also a main effect of sleep (BF_10_ = 4.17% ± 102.32%) and a strong interaction between sleep and trial type (BF_10_ = 24.60% ± 102.75%); however, evidence for follow-up pairwise comparisons revealed no meaningful differences between conditions (BF_10_’s ≤ 2.29). Other main effects and interactions reflected evidence for the null hypothesis (BF_10_’s ≤ 0.003). For burst gradient, there was a strong main effect of threat (BF_10_ = 94.60% ± 2.00%). Burst gradient was greater in the threat than in the no-threat condition. There also was an interaction between trial type and sleep (BF_10_ = 5.10% ± 88.78%); however, follow-up pairwise comparisons revealed no meaningful differences between conditions (BF_10_’s ≤ 1.02). Other main and interaction effects either remained inconclusive or reflected evidence for the null hypothesis (BF_10_’s ≤ 1.51).

## Discussion

The present study aimed to elucidate the effects of sleep deprivation on response inhibition under threat and no-threat conditions and determine effect thresholds by systematically manipulating the magnitude of participants’ sleep deficit (i.e. one night of 0, 2, 4, and 8 hours of sleep opportunity). Results indicated impaired response inhibition following a single night of 2 hours sleep or less, improved response inhibition in the context of performance-contingent threat, but no evidence for reduced control over emotional (threat-related) responding after sleep deprivation. The present findings provide novel insight into sleep deprivation and contextual factors (e.g. selective stopping) that pose meaningful risks to individuals’ inhibitory performance.

### Sleep deprivation impairs behavioral responding and response inhibition

In line with our first hypothesis, the effects of insufficient sleep on behavioral going and stopping were more pronounced with an increased magnitude of sleep deficit. That is, in line with Van Dongen et al. [34] there was a dose-dependent effect of sleep loss, with higher PVT error rates and lower response speed after 0 and 2 hours compared to 8 hours of sleep, but not after 4 hours of sleep. In addition, PVT error rates were higher after 0 hours than after 2 hours of sleep. These effects were mirrored in participants’ go-trial success rate during the ARI task, which was lower after 0 hours compared to 8 hours of sleep, but not after 2 or 4 hours compared to 8 hours of sleep [23]. Go-trial response times were not meaningfully impacted by sleep deprivation, which indicates that negative effects of sleep loss on behavioral responding may have been driven by attentional lapses (e.g. not noticing the “Go” stimulus) rather than delayed action initiation or slower execution [47]. This observation was further confirmed by our EMG burst analyses, which showed no meaningful evidence for an effect of sleep deprivation on EMG burst-onset, -amplitude or -gradient, associated with successful responding. Indeed, and as suggested by Krause et al. [9], thalamo-cortical activity supporting attentional functioning becomes erratic after severe sleep loss, resulting in episodes where attention is sustained, lost, re-established, and then lost again, leading to associated fluctuations in task performance.

Response inhibition during the ARI task was impaired with insufficient sleep, but only in a selective stopping context. That is, on partial trials, stop-signal delays were longer (stopping speed was slower) after 0 and 2 hours compared to 8 hours of sleep, but not after 4 hours of sleep. This result again confirms the dose-dependent effects of sleep loss [34] and extends it to the domain of response inhibition [23], while also confirming our second hypothesis, which stated that the effects of sleep on response inhibition would be exacerbated in a selective stopping context, owing to the greater attentional and cognitive demand associated with selective as opposed to nonselective stopping [10, 11]. Indeed, the observation that sleep deprivation reduced stopping speed in a selective but not in a nonselective stopping context is in line with results from Chuah et al. [54] and confirms that sleep deprivation impacts cognitive control functions more strongly under conditions of high cognitive load [23]. SI, however, was not meaningfully impacted by sleep deprivation, suggesting that while selective stopping was impaired, insufficient sleep did not cause the underlying inhibitory process to become more or less selective [10].

The current study is the first to examine the effects of sleep deprivation on response inhibition in a selective stopping context. Results indicate that meaningful impairment may be expected after 2 hours of sleep or less, but only in relatively complex (i.e. “selective”) stopping contexts. Bayes factors reflected moderate to very strong effect sizes, suggesting that findings may be considered relatively robust.

### Performance-contingent threat improves stopping speed

In partial support of our third hypothesis, the performance-contingent threat of shock resulted in more potentiated go responses during the ARI task, as was evidenced by increased response-related EMG burst amplitudes and burst rise gradients. Amplified initiation of motor responses may be indicative of increased corticospinal excitation under threat, as suggested by previous studies that implemented transcranial magnetic stimulation [26], and potentially functions to allow fast stimulus-driven responding in stressful situations [24]. Against our hypothesis, however, threat improved rather than worsened participants’ inhibitory efficiency, as was evidenced by shorter stop-signal delays (i.e. faster stopping). Better inhibitory performance with threat goes against neurobiological models which suggest that acute threat prioritizes fast stimulus-driven responding over deliberate, goal-directed action [24, 25]. Empirical evidence, however, reveals a more mixed image and it has been suggested that the effects of threat on response inhibition are likely to vary depending on the implemented task paradigm (e.g. SST, ARI, and Go/NoGo) and manipulation of threat (e.g. low vs. high intensity [55]; task-relevant vs. task-irrelevant [56]; for similar argumentation see [20]). Based on our performance-contingent manipulation, which invoked a negative consequence (electric shock) every time participants responded too slow on (partial) go-trials or erroneously responded on (partial) stop trials, it is likely that threat had a motivational effect and encouraged participants to invest available cognitive resources to maintain or improve task performance (for theoretical accounts see [27, 28]). Indeed, subjective responses to the exit questionnaire confirmed that participants experienced higher levels of task motivation under threat. At a behavioral level, this translated to small decreases in go-response times, which occurred in conjunction with improved (faster) stopping speed, suggesting that when performing under threat, participants may have strategically slowed their go-responses, as much as possible, to facilitate successful stopping [19]. Finally, in a selective stopping context, the observed marginal increase in SI under threat indicates that faster stopping may have been achieved through greater enactment of the hyperdirect as opposed to the indirect pathway [12], thus supporting fast global inhibition at the expense of a delayed response in unstopped effectors [10]. Indeed, recent developments indicate that response inhibition may be enacted in a two-stage process (for review see [57]), whereby an initial pause generates global inhibition during attentional capture of nonselective and selective stop signals [10]. A shift to fast stimulus-driven responding from deliberate prefrontal control in neurobiological models of threat [24] aligns with our results, whereby faster stopping is enabled at the cost of precision. This interpretation, however, is tempered by the fact that the effect of threat on stopping interference was below the critical effect size threshold.

### No evidence of increased emotional response after sleep deprivation

Despite the observed main effects of sleep deprivation and threat, the current study found no evidence for our fourth hypothesis. Based on seminal findings from Yoo et al. [29], we predicted that after sleep deprivation, participants would demonstrate exacerbated behavioral responses to threat, owing to amplified hyper-limbic responding by the amygdala and loss of functional connectivity between the amygdala and medial prefrontal cortex, specifically in emotion-provoking (e.g. threatening) circumstances [9]. Almost without exception, observed effect sizes for the interaction between sleep and threat were BF_10_ < 1 and thus indicated evidence for the null hypothesis, although in some cases effects were BF_10_ > 0.3, indicating that evidence should still be considered inconclusive [52]. In the literature, empirical evidence for increased threat-related behavioral responses after sleep deprivation (or sleep restriction) thus far is scarce, with some studies showing evidence for an effect [31, 32] and other studies showing no effect [58]. In the context of response inhibition, the only previous study on this topic implemented sleep restriction (three nights of 5 vs. 8 hours sleep) and found no evidence for the effect [20]. The current study corroborates this observation and extends it in the context of 24 hours of total sleep deprivation.

Discrepancies in outcomes observed across studies may, to an extent, be accounted for by task differences. That is, Yoo et al. [29] implemented a passive viewing task, in which participants were asked to confirm the extent to which they perceived aversive images that were presented, to be emotionally “negative.” Arguably, such explicit reflection carries the risk of strengthening the emotional response [59]. Subsequent studies, including the current one, had participants perform tasks that imposed a range of cognitive and perceptual-motor demands and which required significant top-down control for participants to guide their actions. Exercising top-down control in the context of task execution activates the frontoparietal “task” network (also referred to as the “executive-control” network [60];), which actively inhibits the influence of the stimulus-driven attentional system [61], and which—to an extent—could mitigate effects of sleep deprivation on emotional reactivity that are observed outside of task execution. Alternatively, it may be that in the context of response inhibition, sleep deprivation, and threat impact behavior via different mechanisms (e.g. cortical vs. subcortical). Future studies that implement neurophysiological data collection techniques are required to examine these hypotheses and should capture sleep-deprived and non-sleep-deprived individuals’ neurophysiological responses to threats in both a task and non-task context.

### Limitations

The current study has several limitations. First, it should be acknowledged that the current study implemented sleep restrictions across a single night only. Effects of sleep restriction on cognitive and neurobehavioral functioning also accumulate over time [34]. In cases where sleep is habitually restricted for a longer period, response inhibition could conceivably be impaired with less sleep restriction (e.g. following a night of 4 hours sleep). Given the scarcity of research on sleep and response inhibition, future studies are warranted to examine the potential cumulative effects of sleep restriction. Second, the implementation of a performance-contingent manipulation of threat may have motivated participants to try and improve their task performance and, by doing so, minimize their exposure to the aversive consequence (i.e. electric shock). Indeed, empirical evidence [56] as well as theoretical accounts of acute threat and performance [27, 28] suggests that the effects of threat on performance may differ depending on the perceived “task-relevance” of the stressor and, relatedly, the perceived degree of behavioral control that the individual has over threat exposure. Future studies, therefore, should contrast the effects of performance-contingent and non-contingent manipulations of threat. Third, a limitation of the current study is that our examination of response inhibition was largely restricted to behavioral measures of going and stopping. To more fully examine the effects of sleep deprivation and threat on response inhibition, it is important that future studies include neurophysiological markers of inhibition (e.g. as obtained through electromyography, electroencephalography, and transcranial magnetic stimulation) [10].

## Conclusion

The current study provides novel insight into the effects of sleep deprivation on response inhibition under performance-contingent threat conditions. Sleep deprivation negatively affected response inhibition. Performance-contingent threat improved the speed of inhibition, possibly due to a prioritizing of stopping. However, the hypothesis that sleep-deprived individuals may have greater difficulty exercising top-down inhibitory control over emotional (threat-related) responses, was not supported. These findings bear implications in terms of the extent of sleep deprivation and specific contextual factors that can be expected to pose meaningful risks to individuals’ inhibitory performance. Sleeping for only two hours or less may impair selective stopping in time-critical situations. From an applied perspective, this implies that sleep deprivation may impair adaptive responding and movement coordination in complex tasks that involve multiple effectors, while performance on more simple tasks is less likely to be impacted. While the current study indicates that impairments may only manifest after sleeping for only 2 hours or less, it is yet to be determined if similar impairments could emerge in situations where mild sleep restriction accumulates over time [34]. Furthermore, in high-risk occupational settings where inhibitory performance failure has adverse consequences for health and well-being, stopping speed may be facilitated under performance-contingent threat, but this may come at the cost of reduced precision in ongoing task execution. Further research is required to investigate the specific conditions under which sleep deprivation does or does not diminish top-down control and increase emotional reactivity under threat.

## Supplementary material

Supplementary material is available at *SLEEP* online.

zsae275_suppl_Supplementary_Figure_S1_Tables_S1-S6

## Data Availability

The data underlying this article cannot be shared publicly due to limitations of the ethical approval. The data will be shared on reasonable request to the corresponding author.
