# Supplementary material for: Tired and out of control? Effects of total and partial sleep deprivation on response inhibition under threat and no-threat conditions
Source: Sleep. 2024 Nov 23;48(3):zsae275. doi: 10.1093/sleep/zsae275 (PMC11893544; doi:10.1093/sleep/zsae275)
Supplement: zsae275_suppl_Supplementary_Figure_S1_Tables_S1-S6 [file zsae275_suppl_supplementary_figure_s1_tables_s1-s6.docx]

**Tired and out of control? Effects of total and partial sleep deprivation on response inhibition under threat and no-threat conditions**

**<<<< Supplementary Material >>>**

Arne Nieuwenhuys^1^, Corey G. Wadsley^2^, Robyn Sullivan^1^, John Cirillo^3^, & Winston D. Byblow^1^

*^1^Movement Neuroscience Laboratory, Department of Exercise Sciences, The University of Auckland, Auckland, New Zealand*

*^2^Department of Human Physiology, University of Oregon, Eugene, USA*

*^3^Discipline of Physiology, The University of Adelaide, Adelaide, Australia*

**Corresponding author:** Dr Arne Nieuwenhuys, Department of Exercise Sciences, The University of Auckland, Private Bag 92019, Auckland 1142, New Zealand. Tel: +64 9 923 7974, Email: [a.nieuwenhuys@auckland.ac.nz](mailto:a.nieuwenhuys@auckland.ac.nz)

**Supplementary Results**

**Manipulation Checks**

***Sleep deprivation protocol.*** Data from the sleep deprivation protocol are presented in Tables S1and S2 and Figure S1.

**Table S1.** Means and standard deviations for total sleep time and subjective measures of sleepiness, vigor and affect, before (10pm) and after (8am) the sleep deprivation protocol.

| **Dependent Variable** | | **Sleep Condition** | | | |
| --- | --- | --- | --- | --- | --- |
|  | | **0hr** | **2hr** | **4hr** | **8hr** |
| **TST_subj_ (h:min)** | | 0:00 (0:00) | 1:44 (0:22) | 3:32 (0:38) | 7:22 (0:53) |
| **KSS (1-9)** |  |  |  |  |  |
|  | *before* | 4.27 (2.16) | 4.91 (2.18) | 4.55 (2.22) | 4.81 (2.34) |
|  | *after* | 7.82 (1.65) | 7.32 (1.78) | 5.59 (2.13) | 3.14 (1.39) |
| **Vigor (1-100)** | |  |  |  |  |
|  | *before* | 63.63 (19.15) | 62.76 (19.88) | 61.13 (17.63) | 64.50 (18.26) |
|  | *after* | 27.00 (16.34) | 35.75 (18.59) | 51.25 (16.83) | 73.25 (12.14) |
| **Affect (1-100)** | |  |  |  |  |
|  | *before* | 79.50 (14.90) | 76.38 (15.65) | 75.75 (15.43) | 79.00 (13.61) |
|  | *after* | 68.63 (16.35) | 76.50 (13.34) | 76.38 (13.04) | 80.63 (13.64) |

*Note.* TST = Total Sleep Time; KSS = Karolinska Sleepiness Scale. Values indicate mean (standard deviation).

For total sleep time, there was a strong main effect of sleep (BF_10_ = 2.97×10^54^ ± 0.49%), indicating that all sleep conditions were effectively separated (O_post_’s ≥ 9.63×10^6^). For subjective sleepiness, vigor and affect, before (10pm) and after (8am) the sleep deprivation protocol, there were strong interaction effects between sleep and time (BF_10_ = 5.69×10^15^ ± 2.06%; BF_10_ = 4.41×10^13^ ± 1.74%; BF_10_ = 200.91 ± 1.60%). Before the sleep deprivation protocol, KSS and GVA (vigor and affect) scores did not meaningfully differ between conditions (BF_10_ ≤ 0.30). After the sleep deprivation protocol, subjective sleepiness was higher and vigor lower with less sleep, with moderate to strong differences between all conditions (O_post_’s ≥ 6.40), apart from the 0h and 2h conditions, which did not meaningfully differ from each other (O_post_’s ≤ 0.75; see Supplementary Table S1). Subjective affect was lower (more negative) with 0hr sleep than with 8hr sleep (O_post_ = 59.32). Evidence for other comparisons was inconclusive (O_post_’s ≤ 1.38).

**Table S2.** Means and standard deviations for subjective sleep estimates during the week preceding each experimental session.

| **Dependent Variable** | **0hr** | **2hr** | **4hr** | **8hr** | **main effect of Sleep** |
| --- | --- | --- | --- | --- | --- |
|  | M (SD) | M (SD) | M (SD) | M (SD) | BF_10_ |
| **TST(h:min)** | 7:55 (0:42) | 8:01 (0:57) | 7:56 (0:48) | 8:01 (0:41) | 0.108 |
| **Bed time (hh:min)** | 11:44 (1:03) | 11:23 (0:59) | 11:33 (0:59) | 11:24 (1:02) | 1.046 |
| **Wake time (hh:min)** | 8:15 (0:20) | 8:05 (0:19) | 8:11 (0:13) | 8:00 (0:17) | 0.158 |
| **SOL (min)** | 10.83 (10.09) | 13.78 (8.70) | 14.25 (13.00) | 13.34 (9.98) | 0.395 |
| **WASO (min)** | 5.79 (10.47) | 6.92 (8.47) | 6.52 (10.49) | 6.05 (9.79) | 0.096 |
| **SE (%)** | 93.16 (6.04) | 92.45 (5.21) | 91.58 (6.94) | 93.63 (5.66) | 0.199 |
| **SQ (1-5)** | 3.97 (0.54) | 3.88 (0.59) | 3.92 (0.60) | 4.03 (0.64) | 0.473 |

*Note.* Values indicate mean (standard deviation). Subjective estimates reflect averages across 5 nights preceding each experimental session. Data is from n = 15 participants with complete data. TST = total sleep time, SOL = sleep onset latency, WASO = wake after sleep onset, SE = sleep efficiency, SQ = sleep quality. Sleep efficiency was calculated as total sleep time (TST) divided by total time in bed, such that values reflect the percentage of total time in bed spent sleeping. SQ was rated on a 5-point Likert scale with 1 = very poor, 2 = poor, 3 = fair, 4 = good, 5 = very good.

Subjective sleep estimates as recorded during the week preceding each experimental session showed no meaningful main effects of sleep and, in most cases, reflected evidence for the null-hypothesis (see Table S2 for an overview). Bayesian correlation analysis confirmed that neither average total sleep time across preceding weeks nor total sleep time on the night prior to starting the sleep deprivation protocol was meaningfully associated with task performance (all BF_10_’s ≤ 2.042). Effects of sleep as observed in the present study are unlikely to have been influenced by variability in sleep that occurred outside of the experimental sessions.

For PVT error rate and response speed, analyses showed a strong interaction between sleep and time (BF_10_ = 2.32×10^6^ ± 22.21%; BF_10_ = 1.91×10^7^ ± 4.91%). PVT error rate and response speed did not meaningfully differ between sleep conditions at baseline (BF_10_ = 0.08; BF_10_ = 0.11). After the sleep deprivation protocol, however, error rate was higher and response speed was lower with 0hr sleep than with 2hr, 4hr and 8hr sleep (O_post_’s ≥ 14.31; O_post_’s ≥ 27.96), and with 2hr sleep than with 8hr sleep (O_post_ = 3.72; O_post_ = 80.70). Response speed was also lower with 4h sleep than with 8hr sleep (O_post_ = 15.67), whilst this was not the case for error rate (O_post_ = 1.25). For the 0hr, 2hr and 4hr condition, error rate was higher and response speed was lower after the sleep deprivation protocol than before the sleep deprivation protocol. Evidence for other comparisons was inconclusive (O_post_’s ≤ 1.25; see Figure S1a and 1b).

**
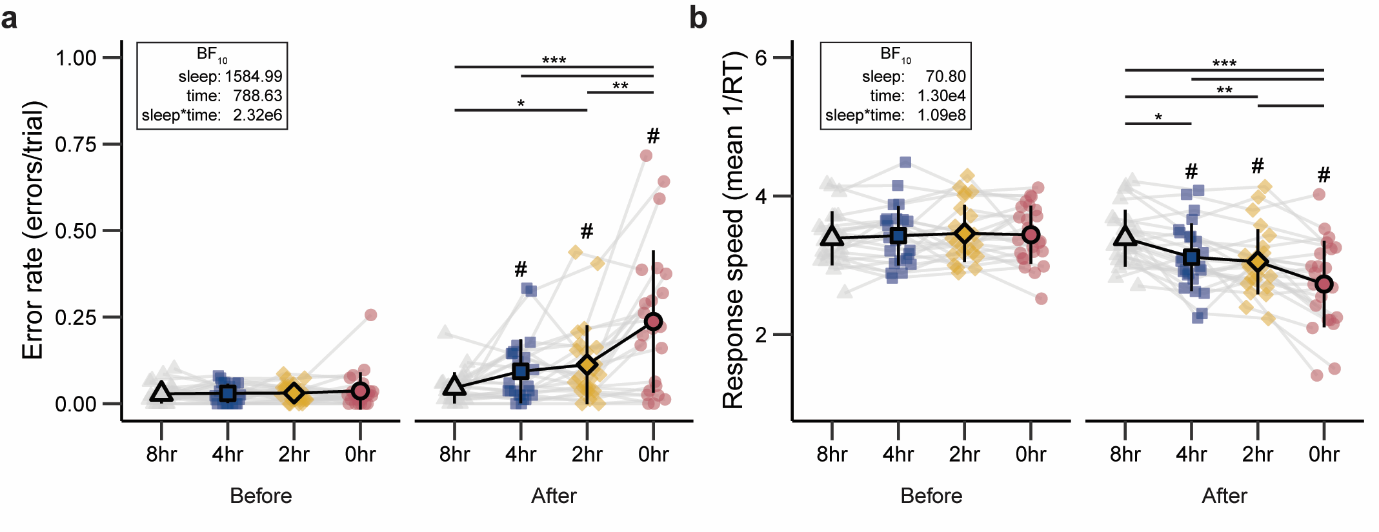
**

**Figure S1.** Psychomotor Vigilance Test (PVT) error rate (**a**) and response speed (**b**) before (10pm) and after (8am) the sleep deprivation protocol, for the 0hr, 2hr, 4hr and 8hr sleep condition. Error bars indicate 95% confidence intervals. *Note.* * = O_post_ ≥ 3; ** = O_post_ ≥ 10; *** = O_post_ ≥ 100, ^#^ = error rate/response speed meaningfully higher/lower after the sleep deprivation protocol than before the sleep deprivation protocol (i.e., O_post_ ≥ 3).

***Manipulation check of threat.*** Data from the exit questionnaire are presented in Table S3. For motivation, valence, and arousal, there were strong main effects of threat (BF_10_ = 1744.28 ± 20.47%; BF_10_ = 976619.04 ± 10.34%; BF_10_ = 11540.99 ± 1.77%). Motivation was higher, valence more negative, and arousal higher, in the Threat than in the No-Threat condition. The main effect of sleep remained inconclusive (0.32 ≤ BF_10_ ≤ 0.70) and the interaction between sleep and threat reflected evidence for the null-hypothesis (0.15 ≤ BF_10_ ≤ 0.26). For dominance scores, no conclusive evidence was observed (BF_10_’s < 2.95).

**Table S3.** Means and standard deviations for all manipulation checks of threat.

| **Dependent Variable** | | **No-Threat** | **Threat** |
| --- | --- | --- | --- |
| **motivation (1-9)***** | |  |  |
|  | *0hr* | 5.67 (2.06) | 7.22 (1.93) |
|  | *2hr* | 5.89 (1.94) | 7.61 (1.24) |
|  | *4hr* | 5.72 (1.90) | 7.89 (1.18) |
|  | *8hr* | 6.44 (1.89) | 7.94 (1.06) |
| **valence (1-9)***** | |  |  |
|  | *0hr* | 3.84 (1.26) | 6.00 (1.11) |
|  | *2hr* | 3.63 (1.71) | 6.26 (1.28) |
|  | *4hr* | 3.63 (1.38) | 5.95 (1.39) |
|  | *8hr* | 3.37 (1.34) | 5.37 (1.21) |
| **arousal (1-9)***** | |  |  |
|  | *0hr* | 6.79 (1.72) | 4.37 (2.11) |
|  | *2hr* | 6.11 (2.05) | 3.84 (1.71) |
|  | *4hr* | 6.42 (1.47) | 3.84 (1.43) |
|  | *8hr* | 5.79 (2.23) | 3.95 (1.62) |
| **dominance (1-9)** | |  |  |
|  | *0hr* | 6.00 (1.67) | 4.79 (1.87) |
|  | *2hr* | 5.16 (1.74) | 4.63 (1.64) |
|  | *4hr* | 6.11 (1.66) | 5.11 (1.82) |
|  | *8hr* | 6.32 (1.70) | 5.37 (1.98) |

*Note.* Motivation (1 = not motivated, 9 = very motivated); Valence (1 = pleasant, 9 = unpleasant); Arousal (1 = excited, 9 = calm); Dominance (1 = controlled, 9 = controlling). Values indicate mean (standard deviation). *** = BF_10_ > 100 (No-Threat vs Threat).

**Behavioural Data**

Behavioral data from the ARI task are presented in Table S4. Outcomes of statistical analyses are reported in the main text.

**Table S4.** Descriptive data for all behavioral variables.

| **Dependent variable** | | | **Sleep condition** | | | |
| --- | --- | --- | --- | --- | --- | --- |
|  |  |  | **0hr** | **2hr** | **4hr** | **8hr** |
| **Go success (%)** | | |  |  |  |  |
|  |  | *No-Threat* | 93.00 (6.35) | 95.39 (4.94) | 96.75 (3.75) | 97.57 (1.99) |
|  |  | *Threat* | 94.15 (6.50) | 97.02 (2.29) | 97.07 (3.10) | 97.77 (1.66) |
| **Go RT_rel_ (ms)** | |  |  |  |  |  |
|  |  | *No-Threat* | 11.72 (6.98) | 11.64 (5.07) | 11.54 (5.94) | 9.94 (5.76) |
|  |  | *Threat* | 13.93 (7.23) | 14.48 (6.63) | 14.50 (6.70) | 12.89 (7.47) |
| **Stopping success (%)** | | |  |  |  |  |
|  | Stop-all | |  |  |  |  |
|  |  | *No-Threat* | 53.42 (1.28) | 53.27 (1.80) | 53.42 (2.23) | 53.87 (1.80) |
|  |  | *Threat* | 53.57 (1.82) | 53.27 (2.08) | 53.27 (2.08) | 53.57 (1.49) |
|  | Partial | |  |  |  |  |
|  |  | *No-Threat* | 50.97 (2.78) | 51.56 (1.99) | 51.64 (2.03) | 52.53 (1.97) |
|  |  | *Threat* | 51.64 (2.16) | 52.31 (1.53) | 52.46 (2.03) | 52.61 (1.97) |
| **SSD (ms)** | | |  |  |  |  |
|  | Stop-all | |  |  |  |  |
|  |  | *No-Threat* | 204.92 (17.59) | 205.50 (19.87) | 200.83 (18.23) | 195.42 (15.79) |
|  |  | *Threat* | 202 (20.26) | 200.17 (16.08) | 198.83 (16.41) | 195.00 (16.83) |
|  | Partial | |  |  |  |  |
|  |  | *No-Threat* | 234.54 (33.12) | 227.25 (22.62) | 220.83 (23.88) | 210.25 (19.69) |
|  |  | *Threat* | 228.13 (34.50) | 219.63 (20.61) | 216.21 (23.42) | 215.50 (19.85) |
| **SI (ms)** | | |  |  |  |  |
|  |  | No-Threat | 70.72 (24.17) | 75.57 (19.39) | 77.01 (22.83) | 76.00 (22.63) |
|  |  | Threat | 76.54 (21.48) | 82.02 (22.07) | 79.93 (23.57) | 80.27 (18.72) |

*Note.* SSD = Stop-Signal Delay; SI = Stopping Interference. Values indicate mean (standard deviation).

**EMG Data**

Electromyographical data from the ARI task are presented in Table S5. Outcomes of statistical analyses are reported in the main text. As indicated in the main text, complete EMG data from all four experimental sessions was available for 18 out of 24 participants due to technical issues. Post-hoc analyses presented in Table S6 indicate that behavioral results for this subsample are similar to that of the total sample, with all reported main effects of sleep and threat maintained.

**Table S5.** Descriptive data for all electromyographical (EMG) variables.

| **Dependent variable** | | | **Sleep condition** | | | |
| --- | --- | --- | --- | --- | --- | --- |
|  |  |  | **0hr** | **2hr** | **4hr** | **8hr** |
| **Baseline EMG (mV)** | | |  |  |  |  |
|  |  | *No-Threat* | 0.007 (0.004) | 0.006 (0.003) | 0.007 (0.004) | 0.007 (0.005) |
|  |  | *Threat* | 0.007 (0.003) | 0.007 (0.003) | 0.007 (0.004) | 0.008 (0.007) |
| **Burst-onset (ms)** | | |  |  |  |  |
|  | Go-respond | |  |  |  |  |
|  |  | *No-Threat* | -52.63 (7.13) | -51.12 (6.12) | -51.04 (6.51) | -50.04 (5.95) |
|  |  | *Threat* | -51.71 (6.92) | -51.43 (6.11) | -50.16 (6.45) | -49.58 (5.90) |
|  | Partial-respond | |  |  |  |  |
|  |  | *No-Threat* | -50.75 (7.24) | -51.72 (5.29) | -51.95 (8.33) | -51.46 (5.65) |
|  |  | *Threat* | -51.78 (7.45) | -50.69 (4.51) | -50.96 (6.34) | -50.76 (7.68) |
| **Burst-amplitude (mV)** | | |  |  |  |  |
|  | Go-respond | |  |  |  |  |
|  |  | *No-Threat* | 0.39 (0.15) | 0.40 (0.25) | 0.40 (0.25) | 0.39 (0.21) |
|  |  | *Threat* | 0.40 (0.15) | 0.42 (0.27) | 0.41 (0.25) | 0.40 (0.21) |
|  | Partial-respond | |  |  |  |  |
|  |  | *No-Threat* | 0.39 (0.18) | 0.41 (0.26) | 0.41 (0.26) | 0.41 (0.24) |
|  |  | *Threat* | 0.41 (0.18) | 0.45 (0.28) | 0.44 (0.27) | 0.44 (0.26) |
| **Burst-gradient (mV/ms)** | | |  |  |  |  |
|  | Go-respond | |  |  |  |  |
|  |  | *No-Threat* | 0.006 (0.002) | 0.006 (0.004) | 0.006 (0.004) | 0.006 (0.003) |
|  |  | *Threat* | 0.006 (0.002) | 0.007 (0.004) | 0.007 (0.004) | 0.007 (0.003) |
|  | Partial-respond | |  |  |  |  |
|  |  | *No-Threat* | 0.006 (0.003) | 0.007 (0.004) | 0.007 (0.004) | 0.007 (0.004) |
|  |  | *Threat* | 0.006 (0.003) | 0.007 (0.004) | 0.007 (0.004) | 0.007 (0.004) |

*Note.* Values indicate mean (standard deviation).

**Table S6.** Comparison of behavioral analyses for the total sample (n = 24) and EMG subsample (n = 18).

| **Dependent Variable** | | **Main Effects** | | | **Interaction Effects** | | | |
| --- | --- | --- | --- | --- | --- | --- | --- | --- |
|  |  | **Sleep (S)** | **Threat (T)** | **Trial Type (TT)** | **S*T** | **S*TT** | **T*TT** | **S*T*TT** |
|  |  | BF_10_ | BF_10_ | BF_10_ | BF_10_ | BF_10_ | BF_10_ | BF_10_ |
| **Go Success Rate** | |  |  |  |  |  |  |  |
|  | *Total sample* | 718.325*** | 2.460 | - | 0.528 | - | - | - |
|  | *EMG subsample* | 14.680** | 0.780 | - | 0.355 | - | - | - |
| **Go RT_rel_** |  |  |  |  |  |  |  |  |
|  | *Total sample* | 0.296 | 53.013** | - | 0.060 | - | - | - |
|  | *EMG subsample* | 0.417 | 42.430** | - | 0.107 | - | - | - |
| **Stopping Success Rate** | |  |  |  |  |  |  |  |
|  | *Total sample* | 0.932 | 0.367 | 76228.681*** | 0.092 | 0.550 | 0.975 | 0.069 |
|  | *EMG subsample* | 0.550 | 0.780 | 25525.69*** | 0.210 | 0.937 | 0.474 | 0.090 |
| **Stop Signal Delay (SSD)** | |  |  |  |  |  |  |  |
|  | *Total sample* | 35.295** | 16.429 | 5.757×10^7^*** | 0.129 | 16.106** | 0.737 | 0.069 |
|  | *EMG subsample* | 6.820* | 10.031 | 1.175×10^9^*** | 0.141 | 5.971* | 0.388 | 0.115 |
| **Stopping Interference** | |  |  |  |  |  |  |  |
|  | *Total sample* | 0.199 | 2.943 | - | 0.074 | - | - | - |
|  | *EMG subsample* | 0.314 | 1.186 | - | 0.080 | - | - | - |

*Note.* * = BF_10_ > 3; ** = BF_10_ > 10; *** = BF_10_ > 100.
